# Overview of SARS-COV-2 infection at the Butantan Penitentiary Progression Center

**DOI:** 10.11606/s1518-8787.2023057004717

**Published:** 2023-05-11

**Authors:** Soraya Gomes de Amorim Andrade, Fernando Moreira Andrade, Anderson da Silva, Maria Regina Alves Cardoso, Guilherme Rodrigues Ferraz, Eliana Nogueira Castro de Barros, Patrícia Emilia Braga, Milena Martello Cristófalo, Júlia Rabello Guerra Vieira, Carolina de Souza Lopes, Alyne Niyama, Juliana Lima de Carvalho, José Mendes Aldrighi

**Affiliations:** I Universidade de São Paulo Faculdade de Saúde Pública Departamento de Saúde, Ciclos de Vida e Sociedade São Paulo SP Brasil Universidade de São Paulo. Faculdade de Saúde Pública. Departamento de Saúde, Ciclos de Vida e Sociedade. São Paulo, SP, Brasil; II Instituto Butantan São Paulo SP Brasil Instituto Butantan. São Paulo, SP, Brasil; III Universidade de São Paulo Faculdade de Saúde Pública Departamento de Epidemiologia São Paulo SP Brasil Universidade de São Paulo. Faculdade de Saúde Pública. Departamento de Epidemiologia. São Paulo, SP, Brasil; IV Santa Casa de São Paulo Faculdade de Ciências Médicas São Paulo SP Brasil Santa Casa de São Paulo. Faculdade de Ciências Médicas. São Paulo, SP, Brasil; V Centro Universitário São Camilo Faculdade de Medicina São Paulo SP Brasil Centro Universitário São Camilo. Faculdade de Medicina. São Paulo, SP, Brasil

**Keywords:** Prisoners, SARS-CoV-2, COVID-19 Testing, Risk Factors, Prevalence, Prisons

## Abstract

**OBJECTIVE:**

To estimate the prevalence of exposure to the SARS-CoV-2 virus among individuals living in restricted freedom.

**METHODS:**

A seroprevalence survey was carried out with the population of the female penitentiary of the Centro de Progressão Penitenciária (CPP) in Butantan (municipality of São Paulo), between June 24 and August 20, 2020. During this period, according to the Secretariat of Penitentiary Administration (SAP), the positivity of rapid tests among inmates ranged from 65% to 78%. The evaluation method used in the study was the “One Step COVID-19” rapid test (chromatography), from the company Wondfo, also using the RT-PCR method in symptomatic participants to confirm the viral condition. The study population consisted of 879 female inmates and 170 employees of the institution.

**RESULTS:**

The prevalence of total antibodies (IgG/IgM) against the SARS-CoV-2 virus in the total population of 1049 study participants was 6.1%; among the population of 879 inmates,a prevalence of 5.8% was observed, and among the institution’s employees, 7.5%.

**CONCLUSIONS:**

The prevalence of covid-19 at the Butantan CPP was low, which is due to the implementation of simple prevention measures at the institution, such as the use of masks (with appropriate changes), emphasis on hygiene, hand washing and social distancing, in addition to other strategies, such as suspending inmates’ visits from relatives and friends and cutting back on elective medical appointments and outside work.

## INTRODUCTION

In December 2019, the World Health Organization (WHO) was alerted to several cases of atypical pneumonia caused by a new strain of coronavirus, named SARS-CoV-2^1^. As of March 2020, such an infection, called covid-19 (coronavirus disease-2019), was considered pandemic. From that moment on, the world faced dramatic numbers, with more than 544 million cases and more than 6.33 million deaths recorded by June 2022^4^. In the same period, Brazil recorded more than 32.1 million cases and more than 670,000 deaths^5^.

The covid-19 pandemic brought significant challenges to health care, as well as the need for advances in the knowledge of the epidemiology of the disease. Measures to try to contain the spread of SARS-CoV-2 have been adopted around the world, such as restrictions on international travel, implementation of lockdowns, orders for the population to stay at home in certain critical periods of the disease’s incidence, interruption of face-to-face classes, dissemination of a universal hand hygiene guide, physical distancing and use of face masks^6^.

Infection with SARS-CoV-2 can present different clinical pictures, ranging from asymptomatic and oligosymptomatic cases to severe symptomatic cases. If, on the one hand, the occurrence of asymptomatic and oligosymptomatic cases made actions to mitigate transmission more challenging for interrupting the transmission chain, on the other hand, the frequency of severe cases significantly impacted the care capacity of the available health services.

Considering the complexity of diagnosing cases of SARS-CoV-2 infection and the limited resources for this diagnosis, the registered epidemiological curve, based on case reports, did not express the spread of infection. Thus, some investigators estimate underreporting at around 10 to 12 times^7,8^.

Faced with this pandemic scenario, the WHO began to recommend carrying out seroprevalence surveys with the aim of estimating seropositivity and, thus, quantifying the real extent of infection in the general population and in specific populations – with regard to both age and to socioeconomic conditions or other risk factors –, aiming to develop public policies and more effective interventions to face this scourge. That is, knowledge of seroprevalence is strategic information in public health, especially in times of pandemic. Knowing the immunity of the population, guaranteed by infection or even vaccine, allows better regional organization of the health organization. In fact, following this WHO request, in 2020 the Ministry of Health launched a research project with 211,129 Brazilians, in more than 62,000 households in 2,474 municipalities, to estimate the seroprevalence achieved by our population^9^.

Morbidity and mortality from SARS-CoV-2 is related to social determinants of health and the vulnerability of some specific populations. A study carried out in São Paulo, for example, showed higher mortality associated with lower educational and income levels, living in crowded households, and subnormal clusters^12^. In this sense, it is expected that liberty-deprived populations are at risk in pandemic situations. However, considering the transmission of SARS-CoV-2 through the air, prison environments offer an even greater risk of contagion. It is estimated, for example, that a positive case infects up to 10 incarcerated people, while under normal conditions this number should not exceed 2–3 people^13^.

Scientific publications related to the incidence of covid-19, as well as other infectious diseases, in the prison population are scarce, pointing to a possible lack of interest in this topic by the scientific community and public authorities, which may result from the stigma and difficulty of access to that group.

An additional concern for this social segment includes the conditions in prison units in Brazil, with overcrowded and poorly ventilated cells, limited access to water and basic sanitation, and prison units lacking structured health modules, including service rooms scattered wherever there is space available and the frequent movement of inmates through the physical areas of the institutions^14^. These particularities increased their susceptibility to the rapid spread of covid-19, a well-documented fact for such other infectious diseases as influenza, tuberculosis and other respiratory diseases^15,16^. In addition to being a major risk for liberty-deprived people, the high incidence of covid-19 in prisons would serve as a source of infection for the general population, considering the movement of employees and the entire social circle involved^17^. The coronavirus can transit between the bars of the prison system itself, given the transfers of prisoners between multiple establishments^18^. In the state of São Paulo alone, the estimated prison population in 2020 was around 231,287 individuals^19^.

In the context of overcrowding, often present in prisons, it is difficult to avoid overcrowding and maintain enhanced hygiene conditions. This is compounded by the fact that the physical structure of prison units, for the most part, has not been planned for air circulation and access to sunlight. Finally, social isolation becomes even more challenging, since, in addition to new individuals joining the system daily, civil servants transit in different spaces, as they return to their homes after working hours within the units^20,21^.

The prison population is very vulnerable to physical and mental problems, both due to the fragility caused by social isolation itself and the stress of liberty deprivation. In addition, it is much more susceptible to infectious diseases^10^.

Thus, estimating the prevalence of exposure to the SARS-CoV-2 virus in individuals living in restricted environments was essential to assess the speed of spread of the disease, as well as to make decisions that could minimize this spread. In order to assess the seroprevalence of SARS-CoV-2 in prisons, the Butantan Institute carried out a seroepidemiological survey of inmates at the Penitentiary Progression Center (CPP) “Marina Marigo Cardoso de Oliveira” in Butantan located in the municipality of São Paulo, Brazil.

## METHODS

### Study Design

Cross-sectional study carried out between June and September 2020, designed to estimate the prevalence of antibodies against SARS-CoV-2 in the Butantan CPP population.

### Butantan Penitentiary Progression Center

The Butantan CPP is a semi-open female prison whose mission is to house inmates in preparation for social life, before final release after serving their sentence. It receives liberty-deprived women (inmates) from various women’s prisons in the State of São Paulo: State Female Penitentiary (FP); FP of the Capital; FP of Tremembé, FP of Campinas, FP Franco da Rocha, FP of Mogi Guaçu, CPP of São Miguel Paulista, among others. It has the capacity to receive 1,110 women and, at the time of the pandemic, it housed 879 individuals.

The site features a vertical architecture, with four four-story buildings, accessed only by stairs, and a fifth two-story building, where the health management sector is located, on the ground floor. Patients who need further medical care remain in the same building, but on the first floor, adjacent to the health care center.

### Study Population

The study population consisted of inmates residing at the Butantã CPP and employees who worked there during the research, totaling 879 inmates, 159 employees and service providers. At the time the study was carried out, visits were suspended as a measure to prevent contamination with SARS-CoV-2.

### Inclusion Criteria

To be inmates of the establishment, to be employees of the Butantan CPP in the health sector (nursing technicians, nurses, psychologists, social and medical workers); to be administrative employees or penitentiary agents, regardless of age, who showed interest in participating in the study by signing the free and informed consent form (TCLE).

### Exclusion Criteria

Potential participants who show no interest in participating in the study.

### Local Infection Containment Strategies

The population under study is characterized by high turnover, as it includes inmates in a semi-open regime in the final phase of serving their sentence. However, since the beginning of the pandemic, those responsible for the prison system have implemented strategies to contain SARS-CoV-2 infection. Thus, visits from friends and family have been prohibited since April 29, 2020. In addition, the inmates did not go out to work, study or to carry out consultations and elective exams.

In addition, an explanatory lecture was held in the chapel of the prison system, for all employees and representatives of the inmates (approximately 250 people), about the disease, with infection control strategies, including guidance on the importance of using masks, cleaning of hands with gel alcohol, and social isolation. Immediately, the use of a mask was recommended, with changes established at most every three hours, and distancing for all employees and inmates. It should be noted that the masks were produced in the prison itself and provided to everyone.

Pregnant women, elderly women and the chronically ill were placed in an isolated ward close to the health sector, as this was the highest risk group among liberty-deprived people. In addition, all public servants were trained to screen suspected cases. Even in the absence of health workers, everyone knew how to screen cases. Inputs were also stocked (alcohol, gloves, masks, goggles and bleach) to ensure that all procedures could be carried out in appropriate conditions, minimizing transmission risks.

The Health Sector and the Butantan CPP Board of Directors also created specific strategies to contain the infection, suspending consultations and elective exams, except in cases of chronically ill and HIV-seropositive patients. In urgent cases, the inmates were sent to hospital and, as soon as they returned, they remained in isolation for fifteen days.

During the pandemic period, two floors, with individual cells, were separated from the common population for suspected cases of covid-19. The symptomatic prisoners were isolated with control of vital data, blood pressure, pulse and oxygen saturation. In the event of hemodynamic instability and drop in oxygen saturation, they were promptly referred to external emergency services. After returning to the prison system, they remained in isolation for 15 days in an individual cell.

Health and administrative employees and penitentiary agents were subjected, still at the entrance, before entering the building, to temperature and oxygen saturation measurement. If there was any abnormality, they were instructed to seek the external health sector. In severe cases, like the inmates, they were sent by ambulance for an external health assessment.

### Participant Selection and Recruitment Strategy

Potential participants from each subgroup were invited to participate in the study by the research physicians responsible for the institution’s health management center. All study procedures were performed at the CPP itself.

### Biological Material Collection

After signing the TCLE, participants were submitted to biological sampling: blood for the rapid serological test, for all participants, and secretion from the nasopharynx and oropharynx using a swab for carrying out the RT-PCR test, only for individuals who reported symptoms at the time of the survey. The inmates were tested following an organized flow through the four buildings and through the four floors.

The type of sample collected varied depending on the subgroup to which the participant was included in the study. Asymptomatic participants had a blood sample collected by venipuncture (with serum separation) for use in the rapid test. Participants presenting any symptoms suspected of infection were assigned to the symptomatic subgroup and had the material for nasopharyngeal or oropharyngeal swabs measured for RT-PCR analysis, in addition to blood sampling.

The collected blood samples were reserved for a minimum of 30–45 minutes at room temperature to be analyzed. If they were not processed immediately, after 45 minutes, the samples were kept under refrigeration at 2–8°C for a maximum of 72 hours following collection, when they were then evaluated.

The secretion samples collected with the swab were stored for up to 72 hours under refrigeration, at 2–8°C, until processing and analysis. If these samples were not processed within 72 hours, they were kept frozen at -70°C until processing and analysis.

### One Step Covid-19 Rapid Test (RST)

The rapid test provided for the study, based on Technical Opinion 03/2020 – DHEM/DIR GENERAL/HCF, was used to perform serological screening in suspected cases of SARS-CoV-2 infection. Positive results indicate that there was previous exposure of the participant to the SARS-CoV-2 virus, but negative results do not rule out asymptomatic infection or previous contact with the virus after long periods of time. The test has a sensitivity of 98.11% and a specificity of 99.72%, according to the data in the test package insert^22^. The processing of the collected samples was carried out at the Covid-19 Diagnostics Laboratory at the Butantan Institute.

### RT-PCR Real Time

RT-PCR performance followed the execution protocol used at the Butantan Institute for the diagnosis of SARS-CoV-2 infection^23^.

### Data Logging, Data Management and Data Analysis Software

Interviews were carried out with the participants with the collection of identification, sociodemographic and clinical data, recorded in a standardized form on the same day of biological sampling. These data, as well as test results, were fed into an electronic information capture system (Epidata, version 1.1.2 of January 2006) specifically designed for this survey. All participants received reports of individual results and were instructed individually according to the result obtained. The management of the database and the analysis of the results was carried out by the Center for Clinical Safety and Risk Management at the Butantan Institute.

### Statistical Analysis

Data from this study were analyzed using the statistical program Stata version 13.0 (StataCorp LP, CollegeStation, Texas, USA).

In the descriptive analysis of sociodemographic, clinical and laboratory variables, the prevalence of antibodies against SARS-CoV-2 and their respective 95% confidence intervals (95%CI) were calculated for each subgroup of the study population (inmates and employees). The chi-square test was used to test the association between the subgroups of the studied population and the prevalence of antibodies against SARS-CoV-2, with a statistical significance level of 5%.

### Ethical Considerations

This study was conducted in full compliance with relevant Brazilian and international ethical regulations or guidelines, and was approved by the Ethics Committee for Analysis of Research Projects (CAPPesq) of the Hospital das Clínicas of the Faculty of Medicine of the University of São Paulo (USP).

## RESULTS

Information was collected from 1,038 participants. Among them, 879 inmates and 159 servants of the Butantan CPP. The median age in the general sample was 36 years; among inmates it was 34.3 years and among civil servants 42.2 years. The group of inmates was formed by female participants in its entirety. Among servers, 82.8% were female (Table 1).

**Table 1:** Sociodemographic data and characterization of the sample of inmates and civil servants.

Variables	Study population (n = 1,038)		Inmates		Servants	
	
			(n = 879)		(n = 159)	
		
	n	%	n	%	n	%
Age (years)						
Median (min–max)	36.0 (16.2–74.0)		34.3 (19.1–74.0)		42.2 (16.2–66.7)	
Sex						
Male	29	2.8	0	0	29	18.2
Female	1009	97.2	879	100	130	81.8
Race/Color						
White	402	38.7	297	33.8	105	66
Black	131	12.6	113	12.9	18	11.3
Brown	468	45.1	434	49.4	34	21.4
Yellow	2	0.2	1	0.1	1	0.6
Indigenous	1	0.1	1	0.1	0	0
Unknown	34	3.3	33	3.8	1	0.6

In the general sample, 45.1% were brown, 38.7% white and 12.6% black. Among the inmates, 49.4% were brown, 33.8% were white and 12.9% were black, and among public servants 66% were white, 21.4% brown and 11.3% black (Table 1).

The prevalence of total antibodies against SARS-Cov-2 found in the general study population was 6.1% (95%CI 4.7–7.7). Specifically among inmates, the prevalence was 5.8% (95%CI 4.3–7.6), and among civil servants, 7.5% (95%CI 4.0–12.8).

Among inmates, the prevalence was detected more frequently (12.5% [95%CI 0.3–48.2]) in younger women (< than 20 years). Among women aged 20 to 29 years, the prevalence was 8.1% [95%CI 5.4–12.1]; among those aged 30 to 39 years, 40 to 49 years and 50 to 59 years, it was 6.1%, 2.5% and 3.2%, respectively; among those over 60 years of age, the prevalence was 8.3% [95%CI 0.1–27.0]). Seropositivity was higher among white women (6.15%) than among black and brown women (5.5%).

The prevalence observed among civil servants was also higher at the extremes of age in the sample, being 12.5% (95%CI 1.6–38.3) among civil servants aged 20 to 29 years and 12.8% (95%CI 4,3–27.4) among those aged 50 to 59 years. A higher prevalence was observed in male than in female servants, being 13.8% and 6.2%, respectively. In the white population, the prevalence was 9.5%, and in the black population, 3.8% (Tables 2 and 3).

**Table 2:** Prevalence of covid-19 in the total population and among inmates and civil servants according to age, sex and race/color.

Variables	Total			Inmates			Servants		
		
	n	RST+	Prevalence (95%CI)	n	RST+	Prevalence (95%CI)	n	RST+	Prevalence (95%CI)
Age years)									
< 20	9	1	11.1 (0.3–48.2)	8	1	12.5 (0.3–52.7)	1	0	0.0 (0.0–97.5^a^)
20–29	288	24	8.3 (5.4–12.1)	272	22	8.1 (5.1–12.0)	16	2	12.5 (1.6–38.3)
30–39	365	21	5.8 (3.6–8.7)	314	19	6.1 (3.7–9.3)	51	2	3.9 (0.5–13.5)
40– 49	248	8	3.2 (1.4–6.3)	199	5	2.5 (0.8–5.8)	49	3	6.1 (1.3–16.9)
50–59	101	7	6.9 (2.8–13.8)	62	2	3.2 (0.4–11.2)	39	5	12.8 (4.3–27.4)
≥ 60	27	2	7.4 (0.9–24.3)	24	2	8.3 (0.1–27.0)	3	0	0.0 (0.0–70.8^a^)
Sex									
Male	29	4	13.8 (3.9–31.7)	0	-	-	29	4	13.8 (3.9–31.7)
Female	1009	59	5.8 (4.5–7.5)	879	68	7.7 (6.1–9.7)	130	8	6.2 (2.7–11.8)
Race/Color									
White	402	28	7.0 (4.7–9.9)	297	18	6.1 (3.6–9.4)	105	10	9.5 (4.7–16.8)
Brown/Black	599	32	5.3 (3.7–7.5)	547	30	5.5 (3.7–7.7)	52	2	3.8 (0.5–13.2)
Yellow	2	0	0.0 (0.0–84.2^a^)	1	0	0.0 (0.0–97.5^a^)	1	0	0.0 (0.0–97.5^a^)
Indigenous	1	0	0.0 (0.0–97.52^a^)	1	0	0.0 (0.0–97.5^a^)	0	-	-
Unreported	34	3	8.8 (1.9–23.7)	33	3	9.1 (1.9–24.3)	1	0	0.0 (0.0–97.5^a^)

**TOTAL**	**1,038**	**63**	**6.1 (4.7–7.7)**	**879**	**51**	**5.8 (4.3–7.6)**	**159**	**12**	**7.5 (4.0–12.8)**

RST: rapid serological test.

^a^ One-tailed, 97.5%CI.

**Table 3:** Result of the serological analysis and its distribution in the study population according to its characteristics.

Variables	Total			Inmates			Servants		
		
	Positive RST (n = 63)	Negative RST (n = 975)	p	Positive RST (n = 51)	Negative RST (n = 828)	p	Positive RST (n = 12)	Negative RST (n = 147)	p
	n (%)	n (%)		n (%)	n (%)		n (%)	n (%)	
Age years)			0.188			**0.045**			0.382
Median (min–max)	33.8 (19.1–63.8)	36.1 (16.2–74.0)		31.5 (19.1–63.8)	34.6 (19.1–74.0)			42.0 (16.2–66.7)	
Sex			0.077			IC			0.234^a^
Male	4 (6.3)	25 (2.6)		-	-		4 (33.3)	25 (17.0)	
Female	58 (92.1)	950 (97.4)		51 (100)	828 (100)		8 (66.7)	122 (83.0)	
Race/Color (n = 1,004)			0.303			0.748			0.339^a^
White/Yellow/Indigenous	28 (46.7)	377 (39.9)		18 (37.5)	281 (35.2)		10 (83.3)	96 (65.8)	
Brown/Black	32 (53.3)	567 (60.1)		30 (62.5)	517 (64.8)		2 (16.7)	50 (34.2)	
Symptoms (anytime)			0.45			0.859			0.555^a^
No	44 (69.8)	723 (74.2)		40 (78.4)	658 (79.5)		4 (33.3)	65 (44.2)	
Yes	19 (30.2)	252 (25.8)		11 (21.6)	170 (20.5)		8 (66.7)	82 (55.8)	
Symptoms at time of test (≤ 14 days)			0.885			0.646			0.746^a^
No	51 (81.0)	782 (80.2)		43 (84.3)	677 (81.8)		8 (66.7)	105 (71.4)	
Yes	12 (19.0)	193 (19.8)		8 (15.7)	151 (18.2)		4 (33.3)	42 (28.6)	
Symptom with medical care (n = 662)			0.278			0.105			0.184
No	52 (82.5)	741 (76.0)		45 (88.2)	623 (75.2)		7 (58.3)	118 (80.3)	
Yes	2 (3.2)	18 (1.8)		0	4 (0.5)		2 (16.7)	14 (9.5)	
Unreported	9 (14.3)	216 (22.2)		6 (11.8)	201 (24.3)		3 (25.0)	15 (10.2)	
Symptom arising from hospitalization			0.324			**0.045**			0.339
No	53 (84.1)	740 (75.9)		44 (86.3)	610 (73.7)		9 (75.0)	130 (88.4)	
Yes	0	1 (0.1)		0	0		0	1 (0.7)	
Unreported	10 (15.9)	234 (24.0)		7 (13.7)	218 (26.3)		3 (25.0)	16 (10.9)	

RST: rapid serological test; IC: impossible to calculate.

^a^ Fisher’s exact test.

Table 4 shows the prevalence of different symptoms in the general sample. The presence or absence of symptoms had no direct association with positive RST in the study population. Symptoms associated with positive RST were chills (p = 0.006), wheezing (p = 0.032), chest pain (p < 0.001), nausea/vomiting (p = 0.024), loss of smell (p < 0.001) and loss of taste (p < 0.001). Among inmates, the association between positive RST and symptoms occurred with chest pain (p < 0.001), loss of smell (p = 0.029) and loss of taste (p = 0.018) – there were no cases requiring hospitalization, as the clinical picture presented was mild (p = 0.045). Among civil servants, positive RST was associated with fever ≥ 37.8°C (p = 0.029), wheezing (p = 0.015), loss of smell (p < 0.001) and loss of taste (p < 0.002).

**Table 4:** Symptoms reported among the 1,038 participants who performed the rapid serological test.

Variables	Total (n = 1,038)	Rapid serological test		p

		Positive (n = 63)	Negative (n = 975)	
		
	n (%)	n (%)	n (%)	
Participants with symptoms				
No	767 (73.9)	44 (69.8)	723 (74.2)	0.45
Yes	271 (26.1)	19 (30.2)	252 (25.8)	
Symptoms				
Fever ≥37.8°C	25 (2.4)	4 (6.3)	21 (2.2)	0.060^a^
Chills	27 (2.6)	5 (7.9)	22 (2.3)	0.006
Tiredness	58 (5.6)	5 (7.9)	53 (5.4)	0.402
Muscle pain (myalgia)	38 (3.7)	5 (7.9)	33 (3.4)	0.062
Sore throat	54 (5.2)	6 (9.5)	48 (4.9)	0.111
Cough	73 (7.0)	6 (9.5)	67 (6.9)	0.425
Runny nose (rhinorrhea)	48 (4.6)	6 (9.5)	42 (4.3)	0.056
Shortness of breath (dyspnea)	51 (4.9)	6 (9.5)	45 (4.6)	0.081
Wheezing	12 (1.2)	3 (4.8)	9 (0.9)	0.032^a^
Chest pain	33 (3.2)	9 (14.3)	24 (2.5)	< 0.001
Other respiratory symptoms	8 (0.8)	0 (0.0)	8 (0.8)	> 0.999^a^
Headache	186 (17.9)	14 (22.2)	172 (17.6)	0.358
Nausea/vomiting	19 (1.8)	4 (6.3)	15 (1.5)	0.024^a^
Abdominal pain	12 (1.2)	1 (1.6)	11 (1.1)	0.530^a^
Diarrhea	20 (1.9)	3 (4.8)	17 (1.7)	0.116^a^
Conjunctivitis	4 (0.4)	0 (0.0)	4 (0.4)	> 0.999^a^
Loss of smell	12 (1.2)	6 (9.5)	6 (0.6)	< 0.001
Loss of taste	13 (1.3)	6 (9.5)	7 (0.7)	< 0.001
Skin rash or finger discoloration	0 (0.0)	0 (0.0)	0 (0.0)	NP
Other symptoms^b^	14 (1.3)	1 (1.6)	13 (1.3)	0.586^a^

IC: impossible to calculate.

^a^ Fisher’s exact test.

^b^ Other symptoms (*sic*): anemia, toothache, ear pain, back pain, lumbar pain, back pain, back pain in the middle of the right side, pain in the body, pain in the right flank, pain in the eye, flu, partial hearing loss, twinges in the chest, high blood pressure, tachycardia, stye and dizziness.

## DISCUSSION

The prison population is very vulnerable to physical and mental problems, due to both the fragility caused by social isolation itself and the stress of liberty deprivation, in addition to being much more susceptible to infectious diseases^10,11,24^. The infrastructure of prisons, in general, is conducive to the spread of infectious diseases, mainly respiratory, such as covid-19. During the testing, the inmates were not visited by family and friends, nor were they allowed to leave the prison, but they were still exposed, as agents and employees frequently leave and return from the facilities.

In the history of viral infections, it is worth mentioning that, in 1918, during the Spanish flu, the disease spread rapidly as a result of the inmates’ close confinement and an inability to isolate the sick. In the prison of San Quentin, state of California, United States, half of the 1900 inmates contracted the disease during the first wave of the epidemic; sick calls increased from 150 to 700 daily^24^.

Distancing becomes unfeasible and the risks of infection increase, resulting in serious cases and death. At the Butantan CPP, these two consequences were avoided, since the restriction of visits and hygiene measures were efficiently taken, in time to minimize inmates’ infection during the study^24^.

However, the population under study shows high turnover and displacement, since it includes inmates in a semi-open regime and who are allowed to work outside the walls in the final phase of serving their sentences. However, since the beginning of the pandemic, those responsible for the prison system have implemented general strategies to contain the SARS-CoV-2 infection, such as the ban on visits by friends and family since April 29, 2020. The inmates remained in the establishment, without permission for work or study trips, or even for appointments and elective exams. Only cases of chronically ill and HIV-seropositive patients were treated otherwise.

Among some solutions to mitigate the damage caused by covid-19 in the penitentiary system were the release of inmates at high risk of infection, such as those of advanced age, those convicted of non-violent crimes and those with clinical comorbidities, in addition to releasing prisoners in freedom parole, without serious crimes, with a remaining sentence of less than two years, always in an attempt to reduce the establishment’s population^25^.

As for the health, administrative and penitentiary staff, they were all submitted, still at the entrance, before entering the building, to temperature and oxygen saturation measurement. In cases of abnormality, they were instructed to seek the external health sector. In serious cases, just like the inmates, they were all sent by ambulance for an external assessment of their health.

The major limitation of the study was the testing of penitentiary agents, who can work as day laborers or on duty. The strategy was to carry out tests within the two teams on duty. There was a loss of testing in six employees, removed from the service during the study.

Another limitation concerns the evaluation method for detecting infection: the rapid test. The study did not have a qualified professional to evaluate the result obtained and associate it with the patient’s clinical profile or other laboratory test results. The test in question was not developed for the detection of SARSCoV-2 antigens in human samples, thus, it is a qualitative assay.

The results of this survey indicated a higher serum prevalence (6.1%) among the study population; in individuals with a positive serological test (n = 63), with 69.8% asymptomatic. Regarding the prevalence of total antibodies in the inmate population, the prevalence was 5.8%, and, among civil servants, the prevalence was 7.5%. Differences in prevalence with regard to age group – 12.5% among younger women, aged less than 20 years, and civil servants between 20 and 29 years old (12.5%) compared to civil servants aged 50 to 59 years old (12.8%) – did not appear to be significant. Some population-based studies have shown differences in prevalence rates between men and women, with a higher prevalence for men in New York^26^and Switzerland^27^. However, studies conducted in Brazil^18,19,28,29^, France^30^and the United States^31^did not identify significant gender differences.

The observed results showed that there was no direct association between the presence or absence of symptoms with positive results in the study population. The symptoms most associated with positive cases for the prevalence of the SARS-Cov-2 antibody were chills, wheezing, chest pain, nausea/vomiting, loss of smell and loss of taste. When evaluating reports of symptoms presented by individuals participating in other surveys, with a positive test for SARS-CoV-2, it is observed that the report of anosmia and ageusia are the most frequent, followed by fatigue, cough, myalgia and diarrhea^32,33^. It is known that most people with covid-19 develop mild or moderate symptoms (80%), around 15% develop severe symptoms, and among the symptoms most associated with covid-19, which at the time of the epidemiological survey were more frequent, are fever (83%–99% of patients), cough (59%–82%), fatigue (44%–70%), anorexia (40%–84%), dyspnea (31%–40%) and myalgias (11%–35%)^34^. Other nonspecific symptoms such as sore throat, headache and diarrhea have been reported^35^. Anosmia and ageusia usually appear at the onset of symptoms^36,37^.

A nationwide epidemiological study, carried out with the prison population, during the period from April 14 to August 31, 2020, reported that there were reports of approximately 4,724 confirmed cases of covid-19. Only in the state of São Paulo, about 25.17% of the prison population was contaminated, indicating an alarming growth in the spread of the disease in this population and placing the state as having the highest rate of confirmed cases in an incarcerated population in all of Brazil^19^.

The country complied with the measures proposed by the WHO^21^in relation to the incarcerated population through Recommendation 62/2020 of the National Council of Justice (CNJ)^38^. The Brazilian Society of Family and Community Medicine issued a document highlighting the need for other measures, such as educational actions, combating fake news, individual and collective hygiene, hygiene of environments, provision of information to family members, and cleaning of hygiene material for security professionals, involving prisoners and several penitentiary professionals^39^.

As of May 11, 2020, there were 603 confirmed cases of covid-19 in Brazilian prisons, resulting in 23 deaths^40^. In just 20 days, the numbers jumped from 1 to over 100^41^. It is noteworthy that, of the 603 cases of covid-19 in Brazilian prisons, 444 (74%) were in the Penitentiary Complex of Papuda^42^, in the Federal District, an institution that houses many imprisoned politicians and criminals with greater purchasing power. Such data shows an inequality in the penitentiary system that reproduces that of society in general, in which there is more access to tests for the new coronavirus for people who occupy a privileged social or financial position^43^.

As this cross-sectional study portrayed a moment of the disease among the inmates, possible fluctuations in the number of cases of covid-19 were not recorded, since longitudinal studies would be necessary. Some population-based studies, with Epicovid-19, carried out in Brazil^44^, and REACT2^45^, conducted in the United Kingdom, reported a decrease in the percentage of the population that presents antibodies, as the pandemic progressed and vaccination actions intensified. Differences in results may occur due to the type of serological test used. In these two cited studies, rapid tests were used through lateral flow immunoassay, while in ours, a chemiluminescent immunoassay was used.

Since April 2020, seroprevalence studies have been reported in several countries, including Brazil, China, France, Germany, Iran, Italy, Spain, England, Peru, Chile, Switzerland, Austria, and United States^29^. In Brazil, there are few population-based seroprevalence studies. Only one of them is national in scope, with a sample that includes 133 municipalities distributed in the 27 federative units of Brazil – the Epicovid epidemiological survey – carried out at multiple moments of the pandemic^51^.

The *Covid-19 Newsletter*, from SAP, provides the following data on the results of the rapid test of servers and inmates on the date of our research: in July 2020, 11.90% of positive cases among servers and 65.00% of the inmates; in August 2020, 11.48% positive cases and 68.47% of inmates; in September 2020, 12.73% of civil servants and 78.23% of inmates. It should also be considered that among the system’s servants, 82% were male and 18% female, while among the prison population, 98% were male and 2% female during this period^51^.

The authors of the study interpret that the implementation of simple prevention measures in this institution, such as the use of masks (with appropriate changes), emphasis on hygiene, hand washing and social distancing, in addition to other strategies, such as suspending inmates’ family and friends visits, cuts in elective medical appointments and outside work, led to the low prevalence of covid-19 in the Butantan CPP.
