# Supplementary material for: Overview of SARS-COV-2 infection at the Butantan Penitentiary Progression Center
Source: Rev Saude Publica. 2023 May 11;57(Suppl 1):10s. doi: 10.11606/s1518-8787.2023057004717 (PMC10185309; doi:10.11606/s1518-8787.2023057004717)
Supplement: Translation

# Translation: Panorama da infecção pelo SARS-COV-2 no Centro de Progressão Penitenciária do Butantan

**Authors:** Soraya Gomes de Amorim Andrade, Fernando Moreira Andrade, Anderson da Silva, Maria Regina Alves Cardoso, Guilherme Rodrigues Ferraz, Eliana Nogueira Castro de Barros, Patrícia Emilia Braga, Milena Martello Cristófalo, Júlia Rabello Guerra Vieira, Carolina de Souza Lopes, Alyne Niyama, Juliana Lima de Carvalho, José Mendes Aldrighi

**Affiliations:** I Brasil Universidade de São Paulo. Faculdade de Saúde Pública. Departamento de Saúde, Ciclos de Vida e Sociedade. São Paulo, SP, Brasil; II Brasil Instituto Butantan. São Paulo, SP, Brasil; III Brasil Universidade de São Paulo. Faculdade de Saúde Pública. Departamento de Epidemiologia. São Paulo, SP, Brasil; IV Brasil Santa Casa de São Paulo. Faculdade de Ciências Médicas. São Paulo, SP, Brasil; V Brasil Centro Universitário São Camilo. Faculdade de Medicina. São Paulo, SP, Brasil

**Keywords:** Prisioneiros, SARS-CoV-2, Teste para COVID-19, Fatores de Risco, Prevalência, Prisões

## Abstract

**OBJETIVO:**

Estimar a prevalência da exposição ao vírus SARS-CoV-2 entre indivíduos vivendo em restrição de liberdade.

**MÉTODOS:**

Foi realizado inquérito de soroprevalência com a população da penitenciária feminina do Centro de Progressão Penitenciária (CPP) do Butantan (município de São Paulo), entre 24 de junho e 20 de agosto de 2020. Nesse período, segundo a Secretaria de Administração Penitenciária (SAP), a positividade dos testes rápidos entre detentos variou de 65 a 78%. O método de avaliação utilizado no estudo foi o teste rápido “*One Step COVID-19*” (cromatografia), da empresa Wondfo, empregando-se também o método RT-PCR em participantes sintomáticos para confirmação do quadro viral. A população do estudo foi constituída por 879 reeducandas e 170 funcionários da instituição.

**RESULTADOS:**

A prevalência de anticorpos totais (IgG/IgM) contra o vírus SARS-CoV-2 na população total de 1.049 participantes do estudo foi de 6,1%; entre a população de 879 reeducandas foi observada prevalência de 5,8% e entre os servidores da instituição, 7,5%.

**CONCLUSÃO:**

Houve baixa prevalência de covid-19 no CPP do Butantan, o que se deve à implementação de medidas de prevenção simples na instituição, como o uso de máscaras (com trocas adequadas), ênfase na higiene, lavagem das mãos e distanciamento social, além de outras estratégias, como suspensão de visitas de familiares e amigos das reeducandas, cortes de consultas médicas eletivas e do trabalho externo.

## INTRODUÇÃO

Em dezembro de 2019, a Organização Mundial da Saúde (OMS) foi alertada sobre vários casos de pneumonia atípica causada por uma nova cepa de coronavírus, nominada SARS-CoV-2^1^. A partir de março de 2020, tal infecção, chamada de covid-19 (*coronavirus disease*-2019), foi considerada pandêmica. A partir desse momento, o mundo se deparou com números dramáticos, sendo contabilizados mais de 544 milhões de casos e mais de 6,33 milhões de óbitos até junho de 2022^4^. No mesmo período, o Brasil registrou mais de 32,1 milhões de casos e mais de 670 mil óbitos^5^.

A pandemia da covid-19 trouxe expressivos desafios à assistência em saúde, bem como a necessidade de avanços no conhecimento da epidemiologia da doença. Medidas para tentar conter o avanço da disseminação do SARS-CoV-2 foram adotadas em todo o mundo, como restrições a viagens internacionais, implementação de *lockdowns*, ordens para a população ficar em casa em determinados períodos críticos da incidência da doença, interrupção de aulas presenciais, divulgação de um guia universal de higienização das mãos, distanciamento físico e uso de máscaras faciais^6^.

A infecção pelo SARS-CoV-2 pode apresentar quadros clínicos diversos, envolvendo desde casos assintomáticos e oligossintomáticos, até sintomáticos graves. Se, por um lado, a ocorrência de casos assintomáticos e oligossintomáticos tornaram as ações de mitigação da transmissão mais desafiadoras para a interrupção da cadeia de transmissão, por outro, a frequência de casos graves impactou significativamente a capacidade assistencial dos serviços de saúde disponíveis.

Diante da complexidade para diagnosticar os casos de infecção pelo SARS-CoV-2 e da limitação de recursos para esse diagnóstico, a curva epidemiológica registrada, baseada na notificação de casos, não expressava a disseminação da infecção. Assim, alguns investigadores estimam subnotificação em cerca de 10 a 12 vezes^7,8^.

Diante desse cenário de pandemia, a OMS passou a recomendar a realização de inquéritos de soroprevalência com objetivo de estimar a soropositividade e, dessa forma, quantificar a real extensão da infecção na população geral e em populações específicas –com relação tanto à idade, como também a condições socioeconômicas ou a outros fatores de risco –, visando elaborar políticas públicas e intervenções mais eficazes para o enfrentamento desse flagelo. Ou seja, o conhecimento da soroprevalência é uma informação estratégica em saúde pública, especialmente em momentos de pandemia. Conhecer a imunidade da população, garantida por infecção ou mesmo vacina, permite melhor organização regional da organização em saúde. Inclusive, seguindo essa solicitação da OMS, o Ministério da Saúde lançou em 2020 um projeto de pesquisa com 211.129 brasileiros, em mais de 62 mil domicílios de 2474 municípios, para estimar a soroprevalência alcançada por nossa população^9-11^.

A morbimortalidade pelo SARS-CoV-2 está relacionada a determinantes sociais em saúde e à vulnerabilidade de algumas populações específicas. Estudo realizado em São Paulo, por exemplo, evidenciou maior mortalidade associada a menor nível educacional e de renda, a vida em aglomeração familiar e a aglomerados subnormais^12^. Nesse sentido, é esperado que populações privadas de liberdade se apresentem como de risco em situações pandêmicas. Não obstante, considerando a transmissão do SARS-CoV-2 pelo ar, ambientes prisionais oferecem ainda maior risco quanto ao contágio. Estima-se, por exemplo que um caso positivo contamine até 10 pessoas encarceradas, enquanto em condições habituais esse número não deve passar de 2-3 pessoas^13^

Publicações científicas relacionadas à incidência de covid-19, bem como de outras doenças infecciosas, na população prisional são escassas, apontando para um possível desinteresse por esse tema pela comunidade científica e pelo poder público, o que pode resultar do estigma e da dificuldade de acesso a esse grupo.

Uma preocupação adicional a esse segmento social inclui as condições nas unidades prisionais do Brasil, com celas superlotadas e pouco ventiladas, acesso limitado a água e a saneamento básico e unidades prisionais carentes de módulos de saúde estruturados, contemplando salas de atendimento espalhadas por onde houver espaço disponível e a locomoção frequente dos detentos pelas áreas físicas das instituições^14^. Essas particularidades aumentaram a suscetibilidade destes à rápida disseminação da covid-19, fato bem documentado para outras doenças infecciosas como influenza, tuberculose e outras doenças respiratórias^15,16^. Além de ser um grande risco para as pessoas privadas de liberdade, a alta incidência de covid-19 em presídios serviria de fonte de infecção para a população geral, considerando-se a movimentação de funcionários e todo o círculo social envolvido^17^. O coronavírus pode transitar entre as grades do próprio sistema prisional, haja vista as transferências de detentos entre os múltiplos estabelecimentos^18^. Somente no estado de São Paulo a população prisional estimada em 2020 era cerca de 231.287 indivíduos^19^.

No contexto de superlotação, muitas vezes presentes das prisões, é difícil evitar a aglomeração e manter condições reforçadas de higiene. Soma-se a isso o fato de a estrutura física das unidades prisionais, em grande parte, não ter sido planejada para a circulação de ar e o acesso à luz solar. Por fim, o isolamento social fica ainda mais desafiador, pois, além de novos indivíduos ingressando no sistema diariamente, os servidores transitam em diferentes espaços, já que retornam às suas casas após o expediente dentro das unidades^20,21^

A população carcerária é muito vulnerável a problemas físicos e mentais, tanto pela fragilidade causada pelo próprio isolamento social, quanto pelo estresse de privação da liberdade. Ademais, é muito mais suscetível às doenças infectocontagiosas^10^.

Assim, estimar a prevalência da exposição ao vírus SARS-CoV-2 em indivíduos vivendo em restrição de liberdade foi fundamental para avaliar a velocidade de disseminação da doença, bem como tomar decisões que minimizassem essa propagação. Com o intuito de avaliar a soroprevalência de SARS-CoV-2 em estabelecimentos prisionais, o Instituto Butantan realizou um inquérito soroepidemiológico nas reeducandas do Centro de Progressão Penitenciária (CPP) do Butantan “Marina Marigo Cardoso de Oliveira”, município de São Paulo, Brasil.

## MÉTODOS

### Desenho do Estudo

Estudo transversal realizado entre os meses de junho e setembro de 2020, planejado para estimar a prevalência de anticorpos contra o SARS-CoV-2 na população do CPP do Butantan.

### Centro de Progressão Penitenciária do Butantã

O CPP do Butantan é um presídio feminino semiaberto cuja missão é abrigar detentas em preparo ao convívio social, antes da liberdade final após o cumprimento de pena. Recebe mulheres privadas de liberdade (reeducandas) provenientes de várias prisões femininas do Estado de São Paulo: Penitenciária Feminina (PF) do Estado; PF da Capital; PF de Tremembé, PF de Campinas, PF Franco da Rocha, PF de Mogi Guaçu, CPP de São Miguel Paulista, dentre outras. Tem capacidade para receber 1.110 mulheres e no momento da pandemia acolhia 879 indivíduos.

O local apresenta uma arquitetura vertical, com quatro prédios de quatro andares, acessados somente por escadas, e um quinto prédio de dois andares, onde se localiza o setor de gerenciamento da saúde, no piso térreo. Os pacientes que necessitam de mais cuidados médicos permanecem no mesmo prédio, porém no primeiro andar, adjacente ao centro de cuidados em saúde.

### População de Estudo

A população do estudo constituiu-se das reeducandas residentes do CPP do Butantã e dos funcionários que atuaram lá durante a realização da pesquisa, totalizando 879 reeducandas, 159 funcionários e prestadores de serviços. Na época de realização do estudo, as visitas estavam suspensas como medida de prevenção à contaminação pelo SARS-CoV-2.

### Critérios de Inclusão

Ser reeducandas do estabelecimento, ser funcionários do CPP do Butantan do setor da saúde (técnicos de enfermagem, enfermeiros, psicólogas, assistentes sociais e médica); ser funcionários administrativos ou agentes penitenciários, independentemente da idade, que demonstrassem interesse em participar do estudo por meio da assinatura do termo de consentimento livre e esclarecido (TCLE).

### Critérios de Exclusão

Potenciais participantes que não demonstrem interesse em participar do estudo.

### Estratégias Locais de Contenção da Infecção

A população em estudo caracteriza-se por ser de grande rotatividade, uma vez que inclui reeducandas em regime semiaberto em fase final do cumprimento de pena. No entanto, desde o início da pandemia, os responsáveis pelo sistema carcerário implementaram estratégias para conter a infecção pelo SARS-CoV-2. Assim, foram proibidas as visitas de amigos e familiares desde 29 de abril de 2020. Além disso, as reeducandas não saíram para trabalhar, estudar e nem para realizar consultas e exames eletivos.

Ademais, foi realizada palestra explicativa na capela do sistema prisional, para todos os funcionários e para as representantes da reeducandas (aproximadamente 250 pessoas), sobre a doença, com estratégias de controle da infecção, incluindo orientações quanto à importância do uso de máscaras, limpeza das mãos com álcool em gel e isolamento social. De imediato, recomendou-se o uso de máscara, com trocas estabelecidas no máximo a cada três horas, e distanciamento para todos os funcionários e reeducandas. Ressalta-se que as máscaras foram produzidas no próprio presídio e fornecidas para todos.

Gestantes, idosas e doentes crônicos foram colocados em ala isolada próxima ao setor da saúde por tratar-se de grupo do mais alto risco dentre as pessoas privadas de liberdade. Além disso, todos os servidores foram treinados para realizar a triagem dos casos suspeitos. Mesmo na ausência de servidores da saúde, todos sabiam triar casos. Ainda foi realizado estoque de insumos (álcool, luvas, máscaras, óculos e água sanitária) para garantir que todos os procedimentos pudessem ser feitos em condições adequadas, minimizando riscos de transmissão.

O Setor da Saúde e a Diretoria do CPP do Butantan criaram ainda estratégias especificas de contenção da infecção, suspendendo consultas e exames eletivos, exceto nos casos de doentes crônicos e soropositivos para HIV. Nos casos de urgência, as reeducandas eram encaminhadas ao hospital e, tão logo retornassem, permaneciam em isolamento durante quinze dias.

No período da pandemia dois andares, com celas individuais, foram separados da população comum para casos suspeitos de covid-19. As reeducandas sintomáticas foram isoladas com controle de dados vitais, pressão arterial, pulso e saturação de oxigênio. Nas ocorrências de instabilidade hemodinâmica e queda da saturação de oxigênio, elas eram encaminhadas prontamente para os serviços externos de urgência. Após o retorno ao sistema prisional, permaneciam em isolamento por 15 dias em cela individual.

Funcionários da saúde e administrativos e agentes penitenciários eram submetidos, ainda na portaria, antes da entrada no prédio, à mensuração da temperatura e saturação de oxigênio. Se houvesse qualquer anormalidade, eram orientados a procurar o setor externo de saúde. Nos casos graves, assim como as reeducandas, eram encaminhados de ambulância para avaliação externa de saúde.

### Seleção dos Participantes e Estratégia de Recrutamento

Os potenciais participantes de cada subgrupo foram convidados a participar do estudo pelos médicos pesquisadores responsáveis pelo centro gerenciador de saúde da instituição. Todos os procedimentos do estudo foram realizados no próprio CPP.

### Coleta de Material Biológico

Após assinarem o TCLE, os participantes foram submetidos à coleta de amostra biológica: de sangue para o teste sorológico rápido, para todos os participantes, e de secreção de nasofaringe e orofaringe por meio de *swab* para realização do teste de RT-PCR, apenas para indivíduos que relatassem sintomas no momento do inquérito. As reeducandas foram testadas seguindo um fluxo organizado dos quatro prédios e pelos quatro andares.

O tipo de amostra coletada variou na dependência do subgrupo ao qual o participante fora inserido no estudo. Participantes assintomáticos tiveram a coleta da amostra de sangue por punção venosa (com separação do soro) para uso no teste rápido. Participantes apresentando algum sintoma suspeito de infecção foram alocados no subgrupo de sintomáticos e tiveram o material para swab nasofaríngeo ou orofaríngeo para análise de RT-PCR aferido, além da coleta de sangue.

As amostras de sangue coletadas foram reservadas por tempo mínimo de 30-45 minutos, em temperatura ambiente, para seguirem à análise. Caso não fossem processadas imediatamente, após os 45 minutos, as amostras eram mantidas sob refrigeração de 2°C a 8°C por no máximo 72h após a coleta, quando eram então avaliadas.

As amostras de secreção coletadas com o *swab* eram armazenadas por até 72h sob refrigeração, 2°C a 8°C, até o processamento e análise. Caso essas amostras não fossem processadas dentro de 72h, elas eram ser mantidas congeladas a -70°C até o momento de processamento e análise.

### Teste Rápido One Step covid-19 (TSR)

O teste rápido fornecido para o estudo, com base no Parecer Técnico 03/2020 – DHEM/DIR GERAL/HCF, foi utilizado para a realização da triagem sorológica em casos suspeitos de infecção por SARS-CoV-2. Resultados positivos indicam que houve exposição prévia do participante ao vírus SARS-CoV-2, porém resultados negativos não descartam infecção assintomática ou contato prévio com o vírus após longos períodos de tempo. O teste apresenta sensibilidade de 98,11% e especificidade de 99,72%, de acordo com os dados presentes na bula do teste^22^. O processamento das amostras coletadas foi realizado no Laboratório de Diagnósticos de covid-19 do Instituto Butantan.

### RT-PCR *Real Time*

A realização do RT-PCR seguiu o protocolo de execução em utilização no Instituto Butantan para o diagnóstico da infecção pelo SARS-CoV-2^23^.

### Registro de Dados, Gerenciamento de Dados e Software de Análise de Dados

Foram realizadas entrevistas com os participantes com coleta de dados de identificação, sociodemográficos e clínicos, registrados em formulário padronizado no mesmo dia da coleta das amostras biológicas. Esses dados, bem como os resultados dos testes, foram inseridos em um sistema eletrônico de captação de informações (Epidata, versão 1.1.2 de janeiro de 2006) elaborado especificamente para esse inquérito. Todos os participantes receberam os laudos dos resultados individuais e foram orientados individualmente de acordo com o resultado obtido. O gerenciamento do banco de dados e de análise dos resultados foi realizado pelo Centro de Segurança Clínica e Gestão de Risco do Instituto Butantan.

### Análise Estatística

Os dados oriundos deste estudo foram analisados utilizando o programa estatístico Stata versão 13.0 (StataCorp LP, CollegeStation, Texas, USA).

Na análise descritiva das variáveis sociodemográficas, clínicas e laboratoriais, calcularam-se a prevalência de anticorpos contra o SARS-CoV-2 e seus respectivos intervalos com 95% de confiança (IC95%) para cada subgrupo da população do estudo (reeducandas e funcionários). Foi utilizado o teste qui-quadrado para testar associação entre os subgrupos da população estudada e a prevalência de anticorpos contra o SARS-CoV-2, tendo como nível de significância estatística 5%.

### Considerações Éticas

Este estudo foi conduzido em plena conformidade com os regulamentos ou diretrizes éticas brasileiras e internacionais pertinentes, e foi aprovado pela Comissão de Ética para Análise de Projetos de Pesquisa (CAPPesq) do Hospital das Clínicas da Faculdade de Medicina da Universidade de São Paulo (USP).

## RESULTADOS

Foram coletadas informações de 1.038 participantes. Dentre eles, 879 reeducandas e 159 servidores do CPP do Butantan. A mediana de idade na amostra geral foi de 36 anos; entre as reeducandas foi de 34,3 anos e entre os servidores 42,2 anos. O grupo de reeducandas foi formado por participantes do sexo feminino em sua totalidade. Entre os servidores, 82,8% eram do sexo feminino (Tabela 1).

**Tabela 1:** Dados sociodemográficos e caracterização da amostra de reeducandas e servidores.

Variáveis	População do estudo (n = 1.038)		Reeducandas		Servidores	
	
			(n = 879)		(n = 159)	
		
	n	%	n	%	n	%
Idade (anos)						
Mediana (mín–máx)	36,0 (16,2–74,0)		34,3 (19,1–74,0)		42,2 (16,2–66,7)	
Sexo						
Masculino	29	2,8	0	0	29	18,2
Feminino	1009	97,2	879	100	130	81,8
Raça/Cor						
Branca	402	38,7	297	33,8	105	66
Preta	131	12,6	113	12,9	18	11,3
Parda	468	45,1	434	49,4	34	21,4
Amarela	2	0,2	1	0,1	1	0,6
Indígena	1	0,1	1	0,1	0	0
Ignorado	34	3,3	33	3,8	1	0,6

Na amostra geral, 45,1% eram pardos, 38,7% brancos e 12,6% pretos. Entre as reeducandas, 49,4% eram pardas, 33,8% eram brancas e 12,9% eram pretas, e entre os servidores 66% eram brancos, 21,4% pardos e 11,3% pretos (Tabela 1).

A prevalência de anticorpos totais contra SARS-Cov-2 encontrada na população geral do estudo foi de 6,1% (IC95% 4,7–7,7). Especificamente entre as reeducandas, a prevalência foi de 5,8% (IC95% 4,3–7,6), e entre os servidores, 7,5% (IC95% 4,0–12,8).

Entre as reeducandas, a prevalência foi detectada com maior frequência (12,5% [IC95% 0,3–48,2]) em mulheres mais jovens, menores de 20 anos. Entre as mulheres de 20 a 29 anos a prevalência foi de 8,1% [IC95% 5,4–12,1]; entre as de 30 a 39 anos, 40 a 49 anos e 50 a 59 anos, foi de 6,1%, 2,5% e 3,2 %, respectivamente; entre as maiores de 60 anos a prevalência foi de 8,3% [IC95% 0,1–27,0]). A soropositividade foi maior entre as mulheres brancas (6,15%) do que entre as pretas e pardas (5,5%).

A prevalência observada entre os servidores foi maior também nos extremos de idade da amostra, sendo 12,5% (IC95%1,6–38,3) entre os servidores de 20 a 29 anos e 12,8% (IC95% 4,3–27,4) entre os de 50 a 59 anos. Maior prevalência foi observada entre os servidores do sexo masculino em relação às do sexo feminino, sendo 13,8% e 6,2%, respectivamente. Na população branca, a prevalência foi de 9,5%, e na preta, 3,8% (Tabelas 2 e 3).

**Tabela 2:** Prevalência de covid-19 na população total e entre as reeducandas e servidores de acordo com a idade, sexo e raça/cor.

Variáveis	Total			Reeducandas			Servidores		
		
	n	TSR+	Prevalência (IC95%)	n	TSR+	Prevalência (IC95%)	n	TSR+	Prevalência (IC95%)
Idade (anos)									
< 20	9	1	11,1 (0,3–48,2)	8	1	12,5 (0,3–52,7)	1	0	0,0 (0,0–97,5^a^)
20 a 29	288	24	8,3 (5,4–12,1)	272	22	8,1 (5,1–12,0)	16	2	12,5 (1,6–38,3)
30 a 39	365	21	5,8 (3,6–8,7)	314	19	6,1 (3,7–9,3)	51	2	3,9 (0,5–13,5)
40 a 49	248	8	3,2 (1,4–6,3)	199	5	2,5 (0,8–5,8)	49	3	6,1 (1,3–16,9)
50 a 59	101	7	6,9 (2,8–13,8)	62	2	3,2 (0,4–11,2)	39	5	12,8 (4,3–27,4)
≥ 60	27	2	7,4 (0,9–24,3)	24	2	8,3 (0,1–27,0)	3	0	0,0 (0,0–70,8^a^)
Sexo									
Masculino	29	4	13,8 (3,9–31,7)	0	-	-	29	4	13,8 (3,9–31,7)
Feminino	1009	59	5,8 (4,5–7,5)	879	68	7,7 (6,1–9,7)	130	8	6,2 (2,7–11,8)
Raça/Cor									
Branca	402	28	7,0 (4,7–9,9)	297	18	6,1 (3,6–9,4)	105	10	9,5 (4,7–16,8)
Parda/Preta	599	32	5,3 (3,7–7,5)	547	30	5,5 (3,7–7,7)	52	2	3,8 (0,5–13,2)
Amarela	2	0	0,0 (0,0–84,2^a^)	1	0	0,0 (0,0–97,5^a^)	1	0	0,0 (0,0–97,5^a^)
Indígena	1	0	0,0 (0,0–97,52^a^)	1	0	0,0 (0,0–97,5^a^)	0	-	-
Não informado	34	3	8,8 (1,9–23,7)	33	3	9,1 (1,9–24,3)	1	0	0,0 (0,0–97,5^a^)

**TOTAL**	**1.038**	**63**	**6,1 (4,7–7,7)**	**879**	**51**	**5,8 (4,3–7,6)**	**159**	**12**	**7,5 (4,0–12,8)**

TSR: teste sorológico rápido.

^a^ Unicaudal, IC97,5%.

**Tabela 3:** Resultado da análise sorológica e sua distribuição na população do estudo de acordo com suas características.

Variáveis	Total			Reeducandas			Servidores		
		
	TSR positivo (n = 63)	TSR negativo (n = 975)	p	TSR positivo (n = 51)	TSR negativo (n = 828)	p	TSR positivo (n = 12)	TSR negativo (n = 147)	p
					
	n (%)	n (%)		n (%)	n (%)		n (%)	n (%)	
Idade (anos)			0,188			**0,045**			0,382
Mediana (mín–máx)	33,8 (19,1–63,8)	36,1 (16,2–74,0)		31,5 (19,1–63,8)	34,6 (19,1–74,0)			42,0 (16,2–66,7)	
Sexo			0,077			NP			0,234^a^
Masculino	4 (6,3)	25 (2,6)		-	-		4 (33,3)	25 (17,0)	
Feminino	58 (92,1)	950 (97,4)		51 (100)	828 (100)		8 (66,7)	122 (83,0)	
Raça/Cor (n = 1.004)			0,303			0,748			0,339^a^
Branca/Amarela/Indígena	28 (46,7)	377 (39,9)		18 (37,5)	281 (35,2)		10 (83,3)	96 (65,8)	
Parda/Preta	32 (53,3)	567 (60,1)		30 (62,5)	517 (64,8)		2 (16,7)	50 (34,2)	
Sintomas (qualquer momento)			0,45			0,859			0,555^a^
Não	44 (69,8)	723 (74,2)		40 (78,4)	658 (79,5)		4 (33,3)	65 (44,2)	
Sim	19 (30,2)	252 (25,8)		11 (21,6)	170 (20,5)		8 (66,7)	82 (55,8)	
Sintomas no momento do teste (≤ 14dias)			0,885			0,646			0,746^a^
Não	51 (81,0)	782 (80,2)		43 (84,3)	677 (81,8)		8 (66,7)	105 (71,4)	
Sim	12 (19,0)	193 (19,8)		8 (15,7)	151 (18,2)		4 (33,3)	42 (28,6)	
Sintoma com atenção médica (n = 662)			0,278			0,105			0,184
Não	52 (82,5)	741 (76,0)		45 (88,2)	623 (75,2)		7 (58,3)	118 (80,3)	
Sim	2 (3,2)	18 (1,8)		0	4 (0,5)		2 (16,7)	14 (9,5)	
Não informado	9 (14,3)	216 (22,2)		6 (11,8)	201 (24,3)		3 (25,0)	15 (10,2)	
Sintoma originado em hospitalização			0,324			**0,045**			0,339
Não	53 (84,1)	740 (75,9)		44 (86,3)	610 (73,7)		9 (75,0)	130 (88,4)	
Sim	0	1 (0,1)		0	0		0	1 (0,7)	
Não informado	10 (15,9)	234 (24,0)		7 (13,7)	218 (26,3)		3 (25,0)	16 (10,9)	

TSR: teste sorológico rápido; NP: não foi possível calcular.

^a^ Teste exato de Fisher.

A Tabela 4 mostra a prevalência dos diferentes sintomas na amostra geral. A presença ou ausência de sintomas não teve associação direta relacionada ao TSR positivo entre a população do estudo. Os sintomas associados com o TSR positivo foram calafrios (p = 0,006), chiado (p = 0,032), dor no peito (p < 0,001), náusea/vômitos (p = 0,024), perda de olfato (p < 0,001) e perda de paladar (p < 0,001). Entre as reeducandas, a associação entre TSR positivo e sintomas ocorreu com dor no peito (p < 0,001), perda de olfato (p = 0,029) e perda de paladar (p = 0,018) – não houve casos com necessidade de hospitalização em função do quadro clínico ter se apresentado como leve (p = 0,045). Entre os servidores, o TSR positivo foi associado com febre ≥ 37,8ºC (p = 0,029), chiado (p = 0,015), perda de olfato (p < 0,001) e perda de paladar (p < 0,002).

**Tabela 4:** Sintomas reportados entre os 1.038 participantes que realizaram o teste sorológico rápido.

Variáveis	Total (n = 1.038)	Teste sorológico rápido		p

		Positivo (n = 63)	Negativo (n = 975)	
		
	n (%)	n (%)	n (%)	
Participantes com sintomas				
Não	767 (73,9)	44 (69,8)	723 (74,2)	0,45
Sim	271 (26,1)	19 (30,2)	252 (25,8)	
Sintomas				
Febre ≥37,8°C	25 (2,4)	4 (6,3)	21 (2,2)	0,060^a^
Calafrios	27 (2,6)	5 (7,9)	22 (2,3)	0,006
Cansaço	58 (5,6)	5 (7,9)	53 (5,4)	0,402
Dor muscular (mialgia)	38 (3,7)	5 (7,9)	33 (3,4)	0,062
Dor de garganta	54 (5,2)	6 (9,5)	48 (4,9)	0,111
Tosse	73 (7,0)	6 (9,5)	67 (6,9)	0,425
Nariz escorrendo (rinorreia)	48 (4,6)	6 (9,5)	42 (4,3)	0,056
Falta de ar (dispneia)	51 (4,9)	6 (9,5)	45 (4,6)	0,081
Chiado	12 (1,2)	3 (4,8)	9 (0,9)	0,032^a^
Dor no peito	33 (3,2)	9 (14,3)	24 (2,5)	< 0,001
Outros sintomas respiratórios	8 (0,8)	0 (0,0)	8 (0,8)	> 0,999^a^
Dor de cabeça	186 (17,9)	14 (22,2)	172 (17,6)	0,358
Náusea/vômitos	19 (1,8)	4 (6,3)	15 (1,5)	0,024^a^
Dor abdominal	12 (1,2)	1 (1,6)	11 (1,1)	0,530^a^
Diarreia	20 (1,9)	3 (4,8)	17 (1,7)	0,116^a^
Conjuntivite	4 (0,4)	0 (0,0)	4 (0,4)	> 0,999^a^
Perda de olfato	12 (1,2)	6 (9,5)	6 (0,6)	< 0,001
Perda de paladar	13 (1,3)	6 (9,5)	7 (0,7)	< 0,001
Rash de pele ou descoloração dos dedos	0 (0,0)	0 (0,0)	0 (0,0)	NP
Outros sintomas^b^	14 (1,3)	1 (1,6)	13 (1,3)	0,586^a^

NP: não foi possível calcular.

^a^ Teste exato de Fisher.

^b^ Outros sintomas (*sic*): anemia, dor de dente, dor ouvido, dor na coluna, dor na lombar, dor nas costas, dor nas costas no meio do lado direito, dor no corpo, dor no flanco direito, dor no olho, gripe, perda parcial da audição, pontadas no peito, pressão alta, taquicardia, terçol e tontura.

## DISCUSSÃO

A população carcerária é muito vulnerável a problemas físicos e mentais, tanto pela fragilidade causada pelo próprio isolamento social, quanto pelo estresse de privação da liberdade, além de ser muito mais suscetível às doenças infectocontagiosas^10,11,24^. A infraestrutura dos presídios, em geral, é propícia à propagação de doenças infectocontagiosas, principalmente respiratórias, como a covid-19. Durante a testagem as detentas não receberam visita de familiares e amigos, nem tiveram permissão de sair do presídio, porém ficaram, ainda assim, expostas, pois os agentes e funcionários saem e retornam frequentemente das instalações.

Na história das infecções virais cabe ressaltar que, em 1918, durante a gripe espanhola, a doença se espalhou rapidamente como resultado do confinamento dos internos, com incapacidade de isolamento dos doentes, fazendo com que, na prisão de San Quentin, estado da Califórnia, nos Estados Unidos, metade dos 1.900 presos contraíssem a doença durante a primeira onda da epidemia: 150 infectados, rapidamente passaram para 700 detentos infectados ao dia^24^.

O distanciamento torna-se inviável e os riscos de infecção aumentam, tendo como consequência casos graves e morte. No CPP do Butantan, essas duas consequências foram evitadas, já que a restrição de visitas e as medidas de higiene foram tomadas eficientemente, a tempo de minimizar a infecção das detentas durante a realização do estudo^24^.

Entretanto, a população em estudo apresenta grande rotatividade e deslocamento, uma vez que inclui reeducandas em regime semiaberto e que tem permissão para trabalhar extramuros na fase final do cumprimento de suas penas. No entanto, desde o início da pandemia, os responsáveis pelo sistema carcerário implementaram estratégias gerais para conter a infecção pelo SARS-CoV-2, como a proibição de visitas de amigos e familiares desde 29 de abril de 2020. As reeducandas permaneceram no estabelecimento, sem permissão para as saídas de trabalho ou estudo, ou ainda para consultas e exames eletivos. Apenas casos de doentes crônicos e soropositivos para HIV foram tratados diferenciadamente.

Entre algumas soluções para mitigar danos do covid-19 no sistema penitenciário constaram a liberação de detentos de alto risco de infecção, tais como aqueles com idade avançada, os condenados por crimes não violentos e os portadores de comorbidades clínicas, além de liberar encarcerados em liberdade condicional, sem crimes graves, com pena restante menor do que dois anos, sempre na tentativa de diminuir a população do estabelecimento^25^.

Quanto aos funcionários da saúde, administrativos e agentes penitenciários, todos eram submetidos, ainda na portaria, antes da entrada no prédio, à mensuração da temperatura e saturação de oxigênio. Em casos de anormalidade, eles eram orientados a procurar o setor externo de saúde. Nos casos graves, assim como as reeducandas, todos eram encaminhados, de ambulância, para avaliação externa de sua saúde.

A grande limitação do estudo foi a testagem dos agentes penitenciários, que podem trabalhar como diaristas ou plantonistas. A estratégia foi realizar a testagens dentro das duas equipes de plantão. Houve uma perda de testagem em seis funcionários, afastados do serviço, durante a realização do estudo.

Outra limitação diz respeito ao método de avaliação para detecção da infecção: o teste rápido. O estudo não dispunha de um profissional qualificado para avaliar o resultado obtido e associá-lo ao perfil clínico do paciente ou a outros resultados de exames laboratoriais. O teste em questão não foi desenvolvido para detecção de antígenos de SARSCoV-2 em amostras humanas, assim, trata-se de um ensaio qualitativo.

Os resultados desta pesquisa indicaram uma soroprevalência mais alta (6,1%) entre a população do estudo; nos indivíduos com teste sorológico positivo (n = 63), sendo que 69,8% se apresentavam assintomáticos. Em relação à prevalência de anticorpos totais na população de reeducandas, a prevalência foi de 5,8%, e, entre os servidores, a prevalência foi de 7,5%. As diferenças de prevalência com relação à faixa etária – 12,5% entre as mulheres mais jovens, com idade inferior a 20 anos, e servidores entre 20 e 29 anos (12,5%) comparados aos servidores de 50 a 59 anos (12,8%) – não pareceram ser significativas. Alguns estudos de base populacional demonstraram diferenças nas taxas de prevalência entre homens e mulheres, com maior prevalência para homens em Nova York^26^e na Suíça^27^. Entretanto, estudos realizados no Brasil^18,19,28,29^, na França^30^ e nos Estados Unidos^31^ não identificaram diferenças significativas entre os sexos.

Os resultados observados mostraram que não houve associação direta entre a presença ou ausência de sintomas com os resultados positivos na população do estudo. Os sintomas mais associados com os casos positivos para a prevalência do anticorpo SARS-Cov-2 foram calafrios, chiado, dor no peito, náusea/vômitos, perda de olfato e perda de paladar. Quando se avaliam relatos de sintomas apresentados por indivíduos participantes de outros inquéritos, com teste positivo para SARS-CoV-2, observa-se que o relato de anosmia e ageusia são os mais frequentes, seguidos de fadiga, tosse, mialgia e diarreia^32,33^. Sabe-se que a maioria das pessoas com covid-19 desenvolvem sintomas leves ou moderados (80%), em torno de 15% desenvolvem grave, e entre os sintomas mais associados ao covid-19, que no momento do inquérito epidemiológico eram mais frequentes, estão febre (83–99% dos pacientes), tosse (59%–82%), fadiga (44%–70%), anorexia (40%–84%), dispneia (31%–40%) e mialgias (11%–35%)^34^. Outros sintomas inespecíficos, como dor de garganta, cefaleia e diarreia têm sido relatados^35^. Anosmia e ageusia normalmente são apresentados no início dos sintomas^36,37^.

Um estudo epidemiológico de abrangência nacional, realizado com a população prisional, durante o período de 14 de abril a 31 de agosto de 2020, relatou que houve relatos de cerca de 4.724 casos confirmados de covid-19. Somente no estado de São Paulo, cerca de 25,17% da população prisional se contaminou, indicando crescimento alarmante de propagação da doença nessa população e colocando o estado como detentor da maior taxa de casos confirmados em população isenta de liberdade em todo o Brasil^19^.

O país acatou as medidas propostas pela OMS^21^em relação à população privada de liberdade por meio da Recomendação 62/2020 do Conselho Nacional de Justiça (CNJ)^38^. A Sociedade Brasileira de Medicina de Família e Comunidade emitiu um documento salientando a necessidade de outras medidas, como ações educativas, combate às *fake news*, higiene individual e coletiva, higiene dos ambientes, fornecimento de informações aos familiares e limpeza do material de higiene dos profissionais de segurança, envolvendo prisioneiros e diversos profissionais penitenciários^39^.

Até o dia 11 de maio de 2020, havia 603 casos de covid-19 confirmados em prisões brasileiras, resultando em 23 óbitos^40^. Com o decorrer de apenas 20 dias, os números saltaram de 1 para mais de 100^41^. Chama a atenção que, dos 603 casos de covid-19 em presídios brasileiros, 444 (74%) estavam no Complexo Penitenciário da Papuda^42^, no Distrito Federal, instituição que abriga muitos políticos presos e criminosos de maior poder aquisitivo. O dado evidencia uma desigualdade no sistema penitenciário que reproduz a da sociedade em geral, na qual há mais acesso a testes para o novo coronavírus para as pessoas que ocupam posição social ou financeira privilegiadas^43^.

Como este estudo transversal retratou um momento da doença entre as reeducandas, possíveis oscilações nos números de casos de covid-19 não foram registrados, já que para isso seriam necessários estudos longitudinais. Alguns estudos de base populacional, com o Epicovid-19, realizado no Brasil^44^, e o REACT2^45^, conduzido no Reino Unido, reportaram diminuição do percentual da população que apresenta anticorpos, conforme a pandemia avançou e as ações de vacinação se intensificaram. Diferenças nos resultados podem ocorrer devido ao tipo de teste sorológico utilizado. Nesses dois estudos citados foram utilizados testes rápidos por meio de imunoensaio de fluxo lateral, enquanto no nosso foi empregada imunoiluminescência.

Desde abril de 2020, estudos de soroprevalência têm sido reportados em vários países, incluindo Brasil, China, França, Alemanha, Irã, Itália, Espanha, Inglaterra, Peru, Chile, Suíça, Áustria e Estados Unidos^29^. No Brasil, há poucos estudos de soroprevalência de base populacional. Apenas um deles é de abrangência nacional, com amostra que inclui 133 municípios distribuídos nas 27 unidades federativas do Brasil – o inquérito epidemiológico Epicovid – realizado em múltiplos momentos da pandemia^51^.

O Boletim Informativo Covid-19, da SAP, aponta os seguintes dados sobre os resultados de teste rápido dos servidores e detentos na data da nossa pesquisa: em julho de 2020, 11,90% dos casos positivos entre os servidores e 65,00% dos detentos; em agosto de 2020, 11,48% casos positivos e 68,47% dos detentos; em setembro de 2020, 12,73% dos servidores e 78,23% dos detentos. Deve-se considerar ainda que entre os servidores do sistema 82% eram do sexo masculino e 18% do feminino, enquanto que entre a população carcerária 98% era do masculino e 2% do feminino nesse período^51^.

Os autores do estudo interpretam que a implementação de medidas de prevenção simples, nessa instituição, como o uso de máscaras (com trocas adequadas), ênfase na higiene, lavagem das mãos e distanciamento social, além de outras estratégias, como suspensão de visitas de familiares e amigos das reeducandas, cortes de consultas médicas eletivas e do trabalho externo, levou à baixa prevalência da covid-19 no CPP do Butantan.
